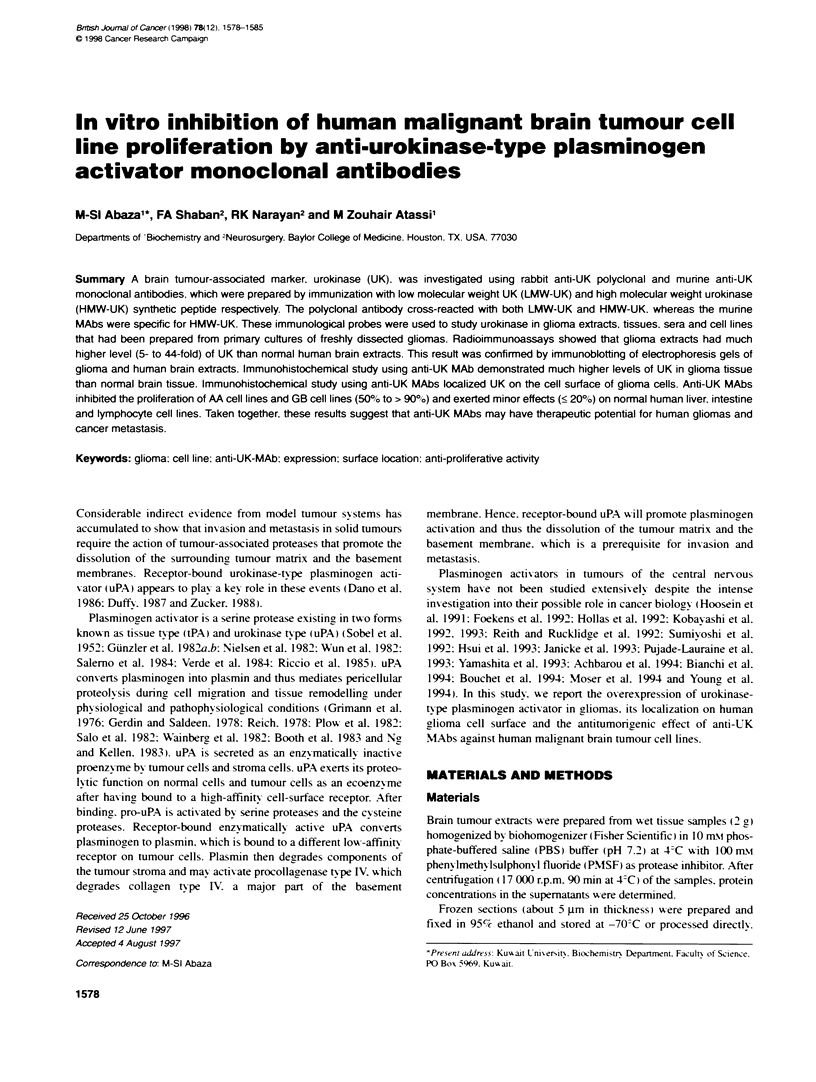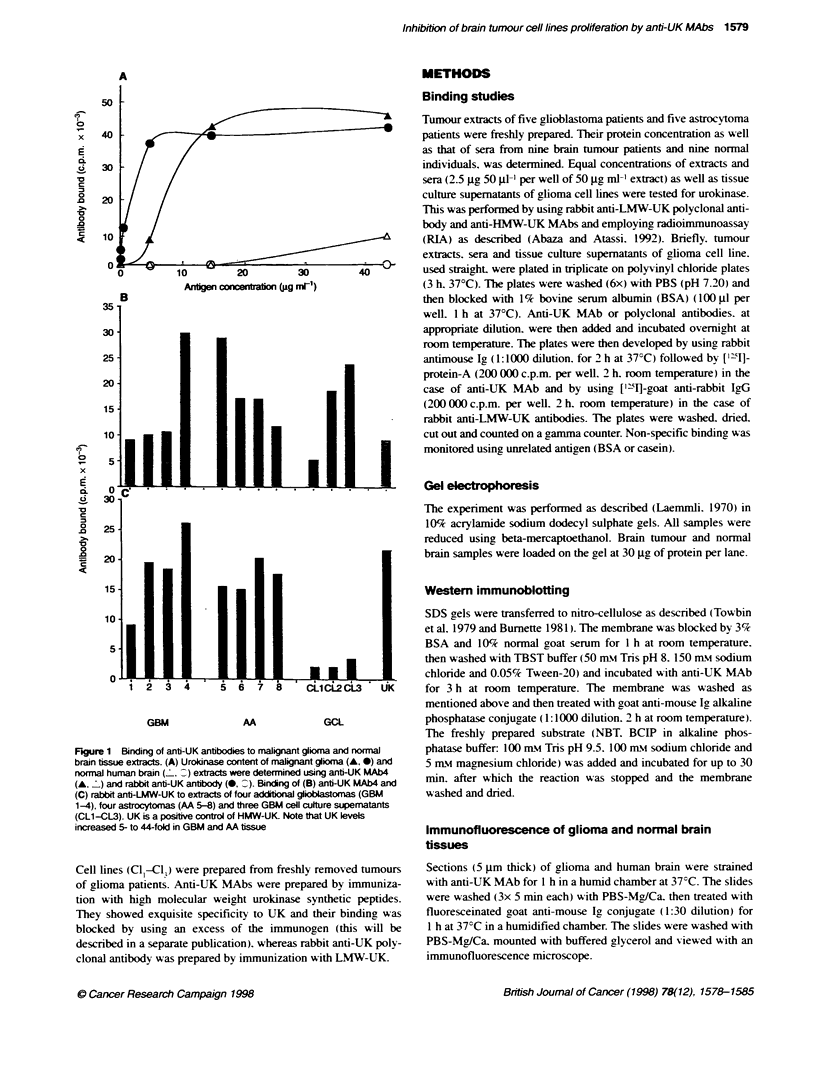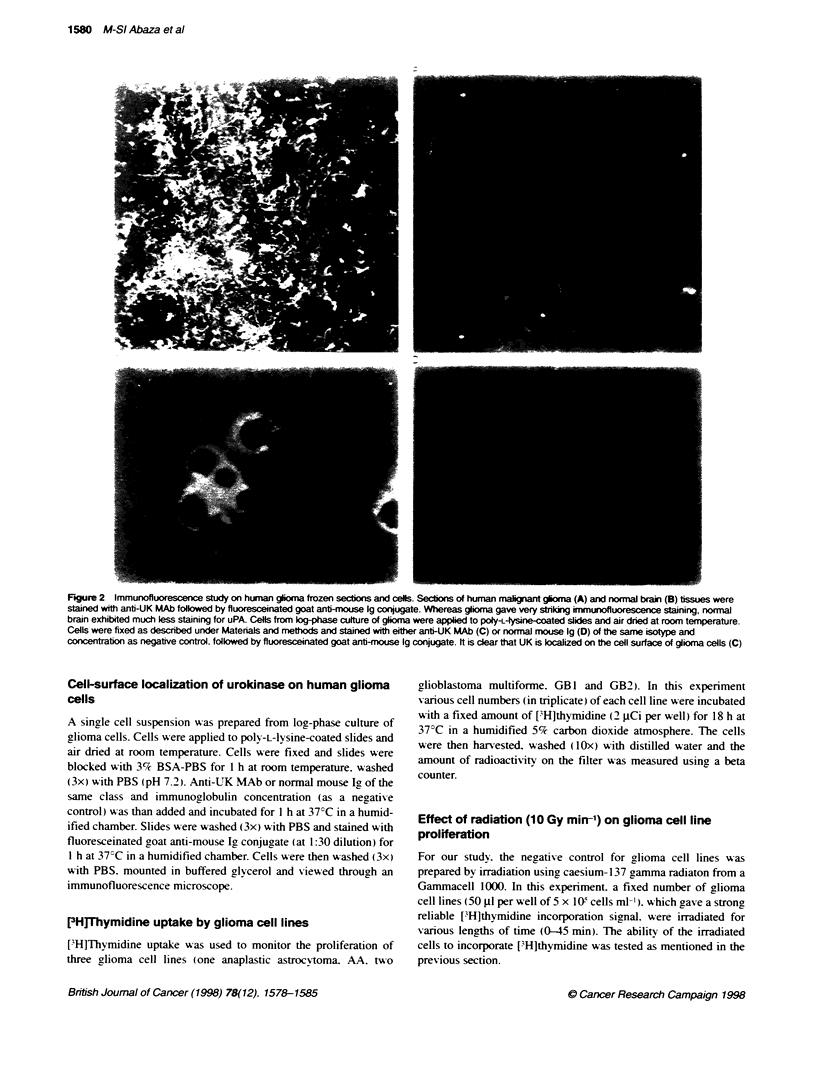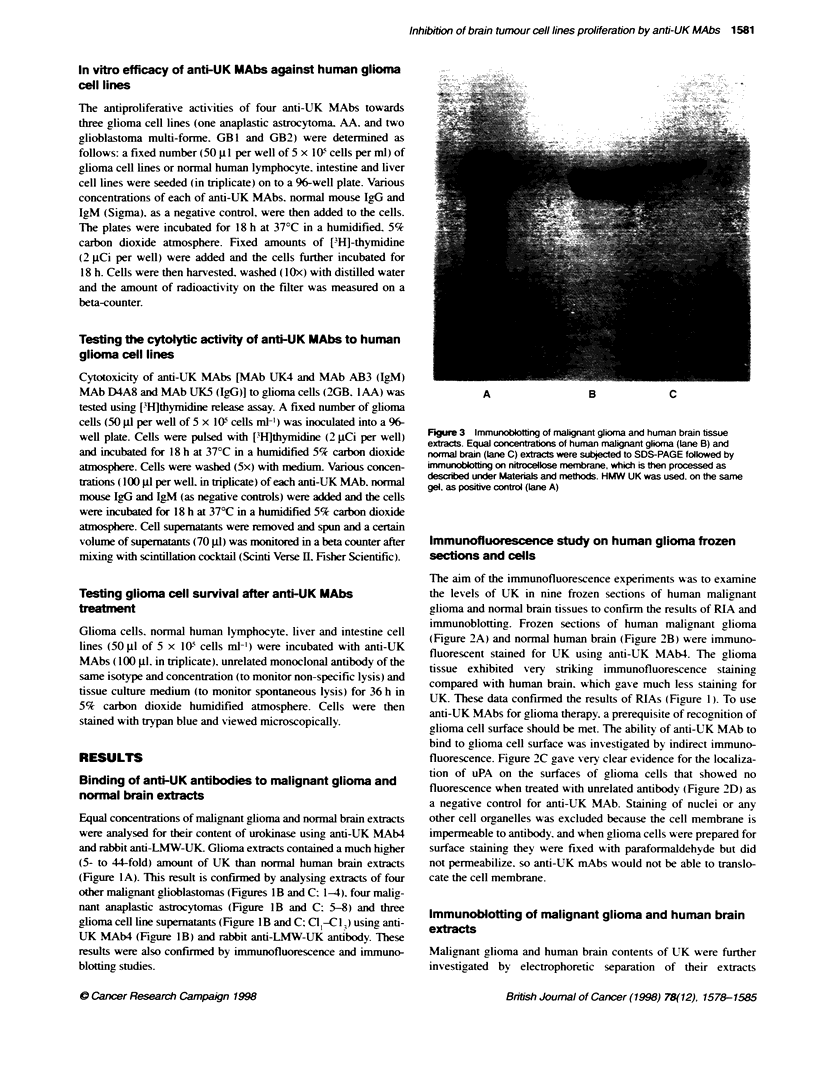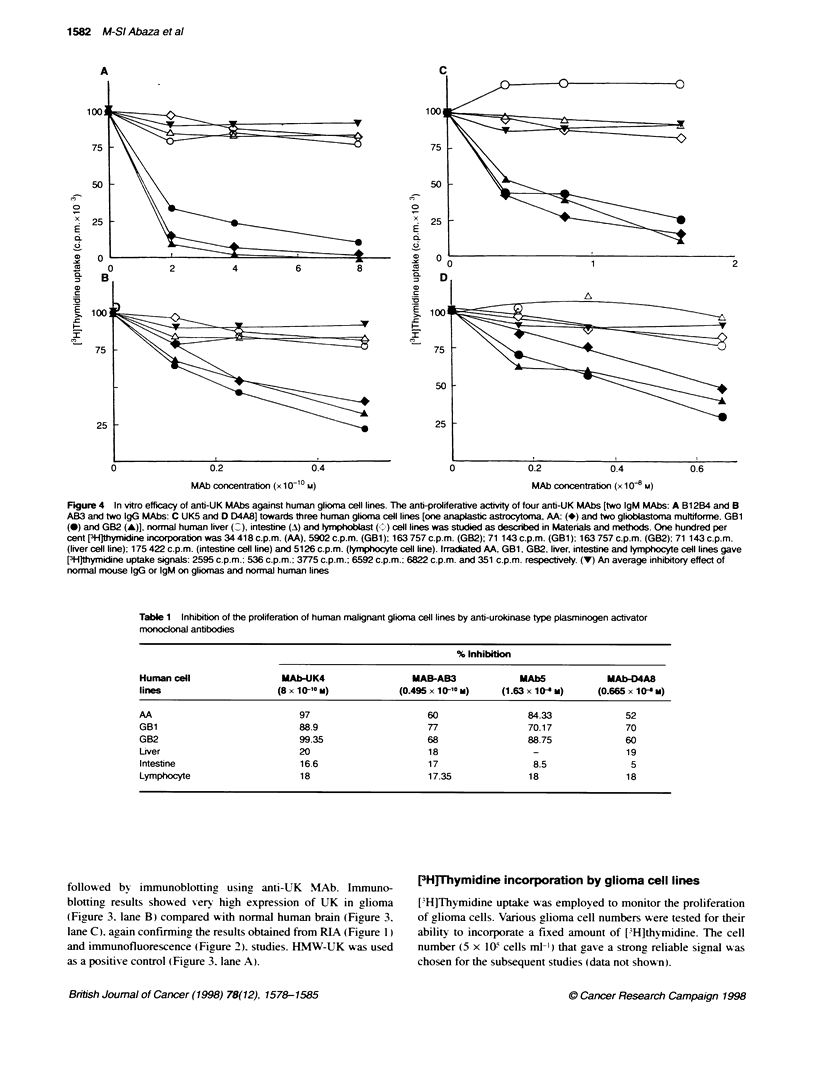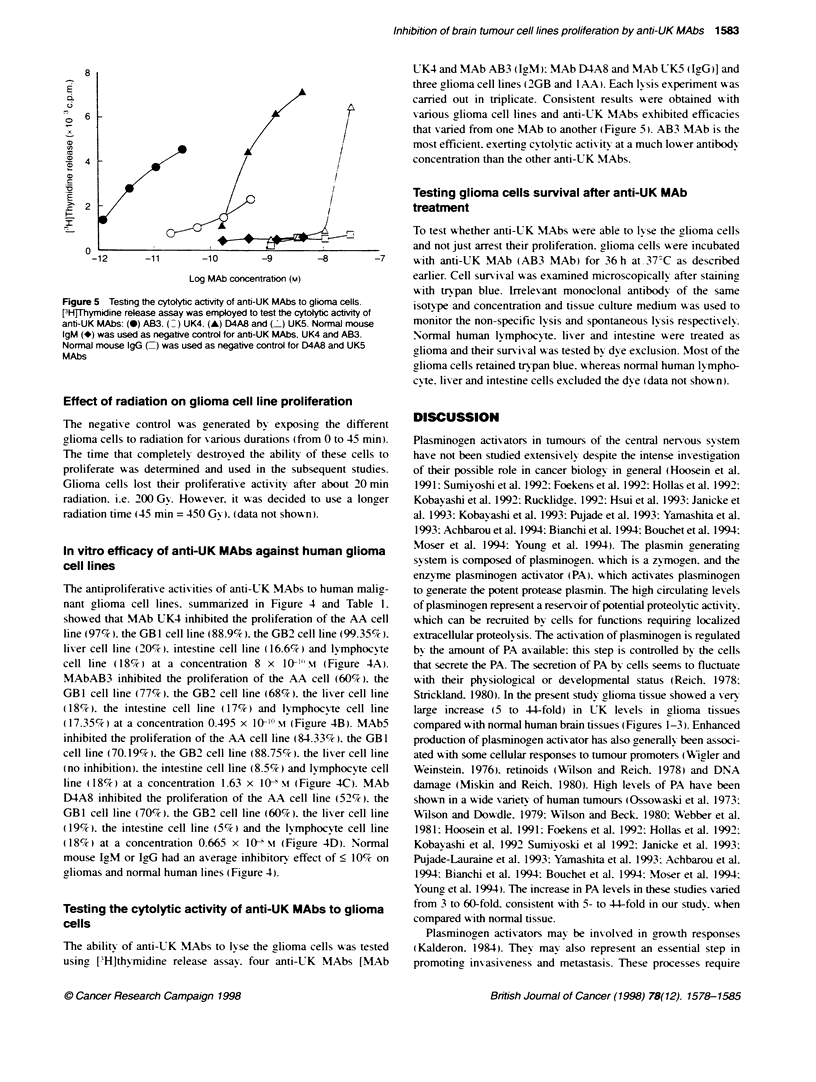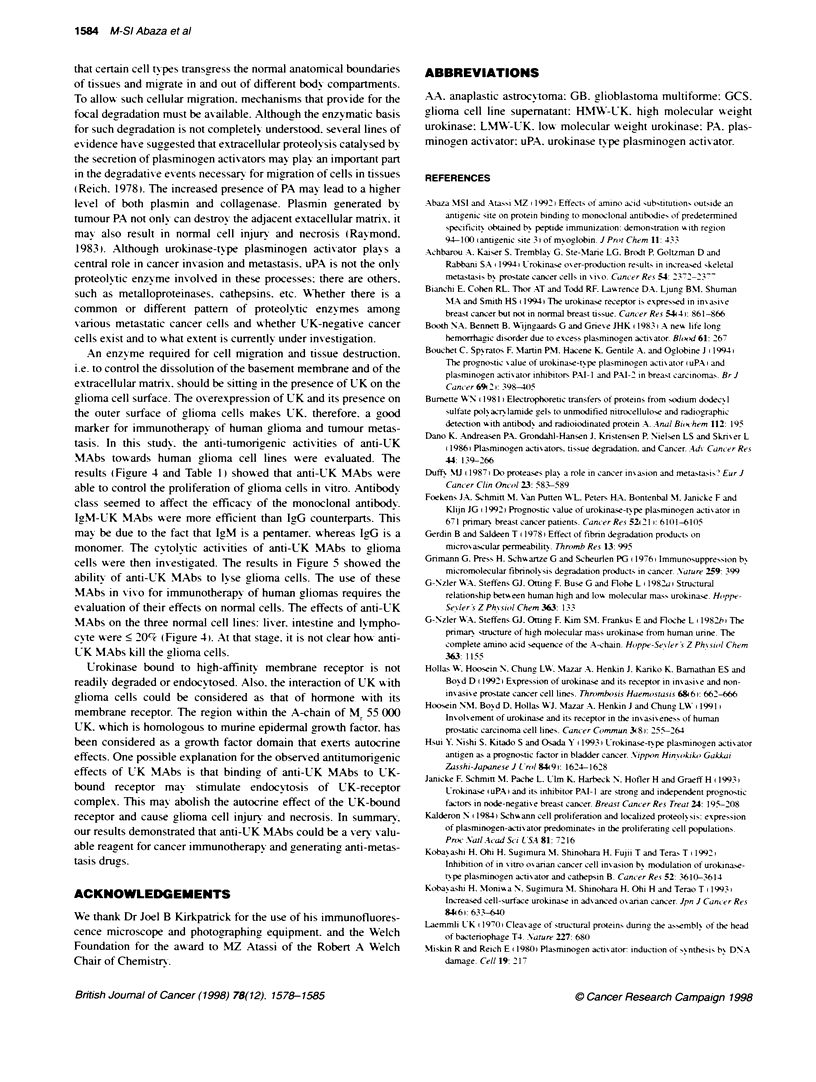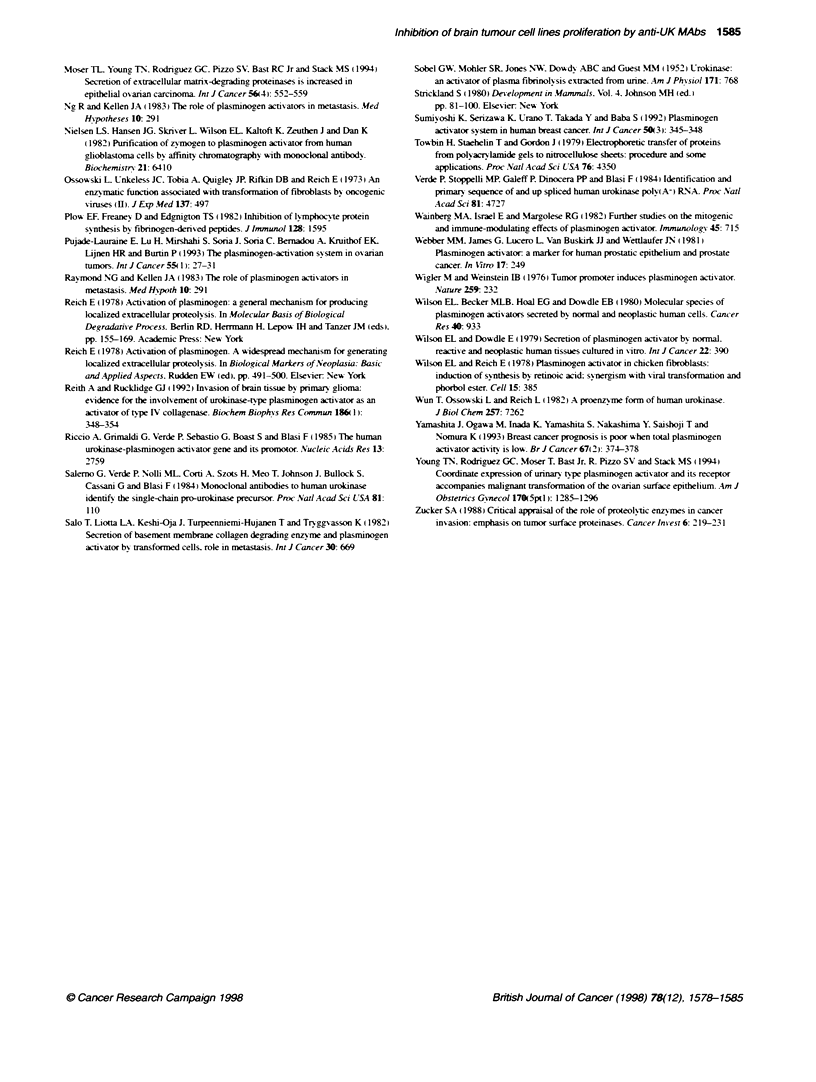# In vitro inhibition of human malignant brain tumour cell line proliferation by anti-urokinase-type plasminogen activator monoclonal antibodies.

**DOI:** 10.1038/bjc.1998.726

**Published:** 1998-12

**Authors:** M. S. Abaza, F. A. Shaban, R. K. Narayan, M. Z. Atassi

**Affiliations:** Department of Biochemistry, Baylor College of Medicine, Houston, TX 77030, USA.

## Abstract

**Images:**


British Journal of Cancer (1998) 78(12). 1578–1585
© 1998 Cancer Research Campaign

### In vitro inhibition of human malignant brain tumour cell line proliferation by anti-urokinase-type plasminogen activator monoclonal antibodies

**M-SI Abaza[1*], FA Shaban[2], RK Narayan[2] and M Zouhair Atassi[1]**

Departments of [1]Biochemistry and [2]Neurosurgery. Baylor College of Medicine. Houston. TX. USA. 77030

**Summary** A brain tumour-associated marker. urokinase (UK). was investigated using rabbit anti-UK polyclonal and murine anti-UK monoclonal antibodies. which were prepared by immunization with low molecular weight UK (LMW-UK) and high molecular weight urokinase (HMW-UK) synthetic peptide respectively. The polyclonal antibody cross-reacted with both LMW-UK and HMW-UK. whereas the murine MAbs were specific for HMW-UK. These immunological probes were used to study urokinase in glioma extracts. tissues. sera and cell lines that had been prepared from primary cultures of freshly dissected gliomas. Radioimmunoassays showed that glioma extracts had much higher level (5- to 44-fold) of UK than normal human brain extracts. This result was confirmed by immunoblotting of electrophoresis gels of glioma and human brain extracts. Immunohistochemical study using anti-UK MAb demonstrated much higher levels of UK in glioma tissue than normal brain tissue. Immunohistochemical study using anti-UK MAbs localized UK on the cell surface of glioma cells. Anti-UK MAbs inhibited the proliferation of AA cell lines and GB cell lines (50% to > 90%) and exerted minor effects (≤ 20%) on normal human liver. intestine and lymphocyte cell lines. Taken together. these results suggest that anti-UK MAbs may have therapeutic potential for human gliomas and cancer metastasis.

**Keywords:** glioma: cell line: anti-UK-MAb: expression: surface location: anti-proliferative activity

Considerable indirect evidence from model tumour systems has accumulated to show that invasion and metastasis in solid tumours require the action of tumour-associated proteases that promote the dissolution of the surrounding tumour matrix and the basement membranes. Receptor-bound urokinase-type plasminogen activator (uPA) appears to play a key role in these events (Dano et al. 1986: Duffy. 1987 and Zucker. 1988).

Plasminogen activator is a serine protease existing in two forms known as tissue type (tPA) and urokinase type (uPA) (Sobel et al. 1952: Günzler et al. 1982*a.b*: Nielsen et al. 1982: Wun et al. 1982: Salerno et al. 1984: Verde et al. 1984: Riccio et al. 1985). uPA converts plasminogen into plasmin and thus mediates pericellular proteolysis during cell migration and tissue remodelling under physiological and pathophysiological conditions (Grimann et al. 1976: Gerdin and Saldeen. 1978: Reich. 1978: Plow et al. 1982: Salo et al. 1982: Wainberg et al. 1982: Booth et al. 1983 and Ng and Kellen. 1983). uPA is secreted as an enzymatically inactive proenzyme by tumour cells and stroma cells. uPA exerts its proteolytic function on normal cells and tumour cells as an ecoenzyme after having bound to a high-affinity cell-surface receptor. After binding. pro-uPA is activated by serine proteases and the cysteine proteases. Receptor-bound enzymatically active uPA converts plasminogen to plasmin. which is bound to a different low-affinity receptor on tumour cells. Plasmin then degrades components of the tumour stroma and may activate procollagenase type IV. which degrades collagen type IV. a major part of the basement membrane. Hence. receptor-bound uPA will promote plasminogen activation and thus the dissolution of the tumour matrix and the basement membrane. which is a prerequisite for invasion and metastasis.

Plasminogen activators in tumours of the central nervous system have not been studied extensively despite the intense investigation into their possible role in cancer biology (Hoosein et al. 1991: Foekens et al. 1992: Hollas et al. 1992: Kobayashi et al. 1992. 1993: Reith and Rucklidge et al. 1992: Sumiyoshi et al. 1992: Hsui et al. 1993: Janicke et al. 1993: Pujade-Lauraine et al. 1993: Yamashita et al. 1993: Achbarou et al. 1994: Bianchi et al. 1994: Bouchet et al. 1994: Moser et al. 1994 and Young et al. 1994). In this study. we report the overexpression of urokinase-type plasminogen activator in gliomas. its localization on human glioma cell surface and the antitumorigenic effect of anti-UK MAbs against human malignant brain tumour cell lines.

*Received 25 October 1996*
*Revised 12 June 1997*
*Accepted 4 August 1997*

*Correspondence to:* M-SI Abaza

### MATERIALS AND METHODS

### Materials

Brain tumour extracts were prepared from wet tissue samples (2 g) homogenized by biohomogenizer (Fisher Scientific) in 10 mM phosphate-buffered saline (PBS) buffer (pH 7.2) at 4°C with 100 mM phenylmethylsulphonyl fluoride (PMSF) as protease inhibitor. After centrifugation (17 000 r.p.m. 90 min at 4°C) of the samples. protein concentrations in the supernatants were determined.

Frozen sections (about 5 μm in thickness) were prepared and fixed in 95% ethanol and stored at –70°C or processed directly.

**Present address*: Kuwait University. Biochemistry Department. Faculty of Science. PO Box 5969. Kuwait.

**Figure 1** Binding of anti-UK antibodies to malignant glioma and normal brain tissue extracts. (**A**) Urokinase content of malignant glioma (▲, ●) and normal human brain (△, ○) extracts were determined using anti-UK MAb4 (▲, △) and rabbit anti-UK antibody (●, ○). Binding of (**B**) anti-UK MAb4 and (**C**) rabbit anti-LMW-UK to extracts of four additional glioblastomas (GBM 1–4), four astrocytomas (AA 5–8) and three GBM cell culture supernatants (CL1–CL3). UK is a positive control of HMW-UK. Note that UK levels increased 5- to 44-fold in GBM and AA tissue

Cell lines ($Cl_1$–$Cl_3$) were prepared from freshly removed tumours of glioma patients. Anti-UK MAbs were prepared by immunization with high molecular weight urokinase synthetic peptides. They showed exquisite specificity to UK and their binding was blocked by using an excess of the immunogen (this will be described in a separate publication), whereas rabbit anti-UK polyclonal antibody was prepared by immunization with LMW-UK.

### METHODS

### Binding studies

Tumour extracts of five glioblastoma patients and five astrocytoma patients were freshly prepared. Their protein concentration as well as that of sera from nine brain tumour patients and nine normal individuals, was determined. Equal concentrations of extracts and sera (2.5 µg 50 µl$^{-1}$ per well of 50 µg ml$^{-1}$ extract) as well as tissue culture supernatants of glioma cell lines were tested for urokinase. This was performed by using rabbit anti-LMW-UK polyclonal antibody and anti-HMW-UK MAbs and employing radioimmunoassay (RIA) as described (Abaza and Atassi, 1992). Briefly, tumour extracts, sera and tissue culture supernatants of glioma cell line, used straight, were plated in triplicate on polyvinyl chloride plates (3 h, 37°C). The plates were washed (6×) with PBS (pH 7.20) and then blocked with 1% bovine serum albumin (BSA) (100 µl per well, 1 h at 37°C). Anti-UK MAb or polyclonal antibodies, at appropriate dilution, were then added and incubated overnight at room temperature. The plates were then developed by using rabbit antimouse Ig (1:1000 dilution, for 2 h at 37°C) followed by [$^{125}$I]-protein-A (200 000 c.p.m. per well, 2 h, room temperature) in the case of anti-UK MAb and by using [$^{125}$I]-goat anti-rabbit IgG (200 000 c.p.m. per well, 2 h, room temperature) in the case of rabbit anti-LMW-UK antibodies. The plates were washed, dried, cut out and counted on a gamma counter. Non-specific binding was monitored using unrelated antigen (BSA or casein).

### Gel electrophoresis

The experiment was performed as described (Laemmli, 1970) in 10% acrylamide sodium dodecyl sulphate gels. All samples were reduced using beta-mercaptoethanol. Brain tumour and normal brain samples were loaded on the gel at 30 µg of protein per lane.

### Western immunoblotting

SDS gels were transferred to nitro-cellulose as described (Towbin et al, 1979 and Burnette 1981). The membrane was blocked by 3% BSA and 10% normal goat serum for 1 h at room temperature, then washed with TBST buffer (50 mM Tris pH 8, 150 mM sodium chloride and 0.05% Tween-20) and incubated with anti-UK MAb for 3 h at room temperature. The membrane was washed as mentioned above and then treated with goat anti-mouse Ig alkaline phosphatase conjugate (1:1000 dilution, 2 h at room temperature). The freshly prepared substrate (NBT, BCIP in alkaline phosphatase buffer: 100 mM Tris pH 9.5, 100 mM sodium chloride and 5 mM magnesium chloride) was added and incubated for up to 30 min, after which the reaction was stopped and the membrane washed and dried.

### Immunofluorescence of glioma and normal brain tissues

Sections (5 µm thick) of glioma and human brain were strained with anti-UK MAb for 1 h in a humid chamber at 37°C. The slides were washed (3× 5 min each) with PBS-Mg/Ca, then treated with fluoresceinated goat anti-mouse Ig conjugate (1:30 dilution) for 1 h at 37°C in a humidified chamber. The slides were washed with PBS-Mg/Ca, mounted with buffered glycerol and viewed with an immunofluorescence microscope.

*© Cancer Research Campaign 1998*

**Figure 2** Immunofluorescence study on human glioma frozen sections and cells. Sections of human malignant glioma (**A**) and normal brain (**B**) tissues were stained with anti-UK MAb followed by fluoresceinated goat anti-mouse Ig conjugate. Whereas glioma gave very striking immunofluorescence staining, normal brain exhibited much less staining for uPA. Cells from log-phase culture of glioma were applied to poly-L-lysine-coated slides and air dried at room temperature. Cells were fixed as described under Materials and methods and stained with either anti-UK MAb (**C**) or normal mouse Ig (**D**) of the same isotype and concentration as negative control, followed by fluoresceinated goat anti-mouse Ig conjugate. It is clear that UK is localized on the cell surface of glioma cells (**C**)

### Cell-surface localization of urokinase on human glioma cells

A single cell suspension was prepared from log-phase culture of glioma cells. Cells were applied to poly-L-lysine-coated slides and air dried at room temperature. Cells were fixed and slides were blocked with 3% BSA-PBS for 1 h at room temperature, washed (3×) with PBS (pH 7.2). Anti-UK MAb or normal mouse Ig of the same class and immunoglobulin concentration (as a negative control) was than added and incubated for 1 h at 37°C in a humidified chamber. Slides were washed (3×) with PBS and stained with fluoresceinated goat anti-mouse Ig conjugate (at 1:30 dilution) for 1 h at 37°C in a humidified chamber. Cells were then washed (3×) with PBS, mounted in buffered glycerol and viewed through an immunofluorescence microscope.

### [$^3$H]Thymidine uptake by glioma cell lines

[$^3$H]Thymidine uptake was used to monitor the proliferation of three glioma cell lines (one anaplastic astrocytoma, AA, two glioblastoma multiforme, GB1 and GB2). In this experiment various cell numbers (in triplicate) of each cell line were incubated with a fixed amount of [$^3$H]thymidine (2 μCi per well) for 18 h at 37°C in a humidified 5% carbon dioxide atmosphere. The cells were then harvested, washed (10×) with distilled water and the amount of radioactivity on the filter was measured using a beta counter.

### Effect of radiation (10 Gy min$^{-1}$) on glioma cell line proliferation

For our study, the negative control for glioma cell lines was prepared by irradiation using caesium-137 gamma radiaton from a Gammacell 1000. In this experiment, a fixed number of glioma cell lines (50 μl per well of $5 \times 10^5$ cells ml$^{-1}$), which gave a strong reliable [$^3$H]thymidine incorporation signal, were irradiated for various lengths of time (0–45 min). The ability of the irradiated cells to incorporate [$^3$H]thymidine was tested as mentioned in the previous section.

© Cancer Research Campaign 1998

### In vitro efficacy of anti-UK MAbs against human glioma cell lines

The antiproliferative activities of four anti-UK MAbs towards three glioma cell lines (one anaplastic astrocytoma, AA, and two glioblastoma multi-forme, GB1 and GB2) were determined as follows: a fixed number (50 µl per well of 5 × $10^5$ cells per ml) of glioma cell lines or normal human lymphocyte, intestine and liver cell lines were seeded (in triplicate) on to a 96-well plate. Various concentrations of each of anti-UK MAbs, normal mouse IgG and IgM (Sigma), as a negative control, were then added to the cells. The plates were incubated for 18 h at 37°C in a humidified, 5% carbon dioxide atmosphere. Fixed amounts of [$^3$H]-thymidine (2 µCi per well) were added and the cells further incubated for 18 h. Cells were then harvested, washed (10×) with distilled water and the amount of radioactivity on the filter was measured on a beta-counter.

### Testing the cytolytic activity of anti-UK MAbs to human glioma cell lines

Cytotoxicity of anti-UK MAbs [MAb UK4 and MAb AB3 (IgM) MAb D4A8 and MAb UK5 (IgG)] to glioma cells (2GB, 1AA) was tested using [$^3$H]thymidine release assay. A fixed number of glioma cells (50 µl per well of 5 × $10^5$ cells $ml^{-1}$) was inoculated into a 96-well plate. Cells were pulsed with [$^3$H]thymidine (2 µCi per well) and incubated for 18 h at 37°C in a humidified 5% carbon dioxide atmosphere. Cells were washed (5×) with medium. Various concentrations (100 µl per well, in triplicate) of each anti-UK MAb, normal mouse IgG and IgM (as negative controls) were added and the cells were incubated for 18 h at 37°C in a humidified 5% carbon dioxide atmosphere. Cell supernatants were removed and spun and a certain volume of supernatants (70 µl) was monitored in a beta counter after mixing with scintillation cocktail (Scinti Verse II, Fisher Scientific).

### Testing glioma cell survival after anti-UK MAbs treatment

Glioma cells, normal human lymphocyte, liver and intestine cell lines (50 µl of 5 × $10^5$ cells $ml^{-1}$) were incubated with anti-UK MAbs (100 µl, in triplicate), unrelated monoclonal antibody of the same isotype and concentration (to monitor non-specific lysis) and tissue culture medium (to monitor spontaneous lysis) for 36 h in 5% carbon dioxide humidified atmosphere. Cells were then stained with trypan blue and viewed microscopically.

### RESULTS

### Binding of anti-UK antibodies to malignant glioma and normal brain extracts

Equal concentrations of malignant glioma and normal brain extracts were analysed for their content of urokinase using anti-UK MAb4 and rabbit anti-LMW-UK. Glioma extracts contained a much higher (5- to 44-fold) amount of UK than normal human brain extracts (Figure 1A). This result is confirmed by analysing extracts of four other malignant glioblastomas (Figures 1B and C: 1–4), four malignant anaplastic astrocytomas (Figure 1B and C: 5–8) and three glioma cell line supernatants (Figure 1B and C: $Cl_1$–$Cl_3$) using anti-UK MAb4 (Figure 1B) and rabbit anti-LMW-UK antibody. These results were also confirmed by immunofluorescence and immunoblotting studies.

**Figure 3** Immunoblotting of malignant glioma and human brain tissue extracts. Equal concentrations of human malignant glioma (lane B) and normal brain (lane C) extracts were subjected to SDS-PAGE followed by immunoblotting on nitrocellose membrane, which is then processed as described under Materials and methods. HMW UK was used, on the same gel, as positive control (lane A)

### Immunofluorescence study on human glioma frozen sections and cells

The aim of the immunofluorescence experiments was to examine the levels of UK in nine frozen sections of human malignant glioma and normal brain tissues to confirm the results of RIA and immunoblotting. Frozen sections of human malignant glioma (Figure 2A) and normal human brain (Figure 2B) were immunofluorescent stained for UK using anti-UK MAb4. The glioma tissue exhibited very striking immunofluorescence staining compared with human brain, which gave much less staining for UK. These data confirmed the results of RIAs (Figure 1). To use anti-UK MAbs for glioma therapy, a prerequisite of recognition of glioma cell surface should be met. The ability of anti-UK MAb to bind to glioma cell surface was investigated by indirect immunofluorescence. Figure 2C gave very clear evidence for the localization of uPA on the surfaces of glioma cells that showed no fluorescence when treated with unrelated antibody (Figure 2D) as a negative control for anti-UK MAb. Staining of nuclei or any other cell organelles was excluded because the cell membrane is impermeable to antibody, and when glioma cells were prepared for surface staining they were fixed with paraformaldehyde but did not permeabilize, so anti-UK mAbs would not be able to translocate the cell membrane.

### Immunoblotting of malignant glioma and human brain extracts

Malignant glioma and human brain contents of UK were further investigated by electrophoretic separation of their extracts

© Cancer Research Campaign 1998

**Figure 4** In vitro efficacy of anti-UK MAbs against human glioma cell lines. The anti-proliferative activity of four anti-UK MAbs [two IgM MAbs: **A** B12B4 and **B** AB3 and two IgG MAbs: **C** UK5 and **D** D4A8] towards three human glioma cell lines [one anaplastic astrocytoma, AA: (◆) and two glioblastoma multiforme, GB1 (●) and GB2 (▲)], normal human liver (○), intestine (△) and lymphoblast (◇) cell lines was studied as described in Materials and methods. One hundred per cent [3H]thymidine incorporation was 34 418 c.p.m. (AA), 5902 c.p.m. (GB1); 163 757 c.p.m. (GB2); 71 143 c.p.m. (GB1); 163 757 c.p.m. (GB2); 71 143 c.p.m. (liver cell line); 175 422 c.p.m. (intestine cell line) and 5126 c.p.m. (lymphocyte cell line). Irradiated AA, GB1, GB2, liver, intestine and lymphocyte cell lines gave [3H]thymidine uptake signals: 2595 c.p.m.; 536 c.p.m.; 3775 c.p.m.; 6592 c.p.m.; 6822 c.p.m. and 351 c.p.m. respectively. (▼) An average inhibitory effect of normal mouse IgG or IgM on gliomas and normal human lines

**Table 1** Inhibition of the proliferation of human malignant glioma cell lines by anti-urokinase type plasminogen activator monoclonal antibodies

| | % Inhibition | | | |
|---|---|---|---|---|
| **Human cell lines** | **MAb-UK4 ($8 \times 10^{-10}$ M)** | **MAB-AB3 ($0.495 \times 10^{-10}$ M)** | **MAb5 ($1.63 \times 10^{-8}$ M)** | **MAb-D4A8 ($0.665 \times 10^{-8}$ M)** |
| AA | 97 | 60 | 84.33 | 52 |
| GB1 | 88.9 | 77 | 70.17 | 70 |
| GB2 | 99.35 | 68 | 88.75 | 60 |
| Liver | 20 | 18 | – | 19 |
| Intestine | 16.6 | 17 | 8.5 | 5 |
| Lymphocyte | 18 | 17.35 | 18 | 18 |

followed by immunoblotting using anti-UK MAb. Immunoblotting results showed very high expression of UK in glioma (Figure 3, lane B) compared with normal human brain (Figure 3, lane C), again confirming the results obtained from RIA (Figure 1) and immunofluorescence (Figure 2), studies. HMW-UK was used as a positive control (Figure 3, lane A).

### [3H]Thymidine incorporation by glioma cell lines

[3H]Thymidine uptake was employed to monitor the proliferation of glioma cells. Various glioma cell numbers were tested for their ability to incorporate a fixed amount of [3H]thymidine. The cell number ($5 \times 10^5$ cells $ml^{-1}$) that gave a strong reliable signal was chosen for the subsequent studies (data not shown).

 © Cancer Research Campaign 1998

**Figure 5** Testing the cytolytic activity of anti-UK MAbs to glioma cells. [$^{3}$H]Thymidine release assay was employed to test the cytolytic activity of anti-UK MAbs: (●) AB3, (○) UK4, (▲) D4A8 and (△) UK5. Normal mouse IgM (◆) was used as negative control for anti-UK MAbs, UK4 and AB3. Normal mouse IgG (□) was used as negative control for D4A8 and UK5 MAbs

### Effect of radiation on glioma cell line proliferation

The negative control was generated by exposing the different glioma cells to radiation for various durations (from 0 to 45 min). The time that completely destroyed the ability of these cells to proliferate was determined and used in the subsequent studies. Glioma cells lost their proliferative activity after about 20 min radiation, i.e. 200 Gy. However, it was decided to use a longer radiation time (45 min = 450 Gy), (data not shown).

### In vitro efficacy of anti-UK MAbs against human glioma cell lines

The antiproliferative activities of anti-UK MAbs to human malignant glioma cell lines, summarized in Figure 4 and Table 1, showed that MAb UK4 inhibited the proliferation of the AA cell line (97%), the GB1 cell line (88.9%), the GB2 cell line (99.35%), liver cell line (20%), intestine cell line (16.6%) and lymphocyte cell line (18%) at a concentration $8 \times 10^{-10}$ M (Figure 4A). MAbAB3 inhibited the proliferation of the AA cell (60%), the GB1 cell line (77%), the GB2 cell line (68%), the liver cell line (18%), the intestine cell line (17%) and lymphocyte cell line (17.35%) at a concentration $0.495 \times 10^{-10}$ M (Figure 4B). MAb5 inhibited the proliferation of the AA cell line (84.33%), the GB1 cell line (70.19%), the GB2 cell line (88.75%), the liver cell line (no inhibition), the intestine cell line (8.5%) and lymphocyte cell line (18%) at a concentration $1.63 \times 10^{-8}$ M (Figure 4C). MAb D4A8 inhibited the proliferation of the AA cell line (52%), the GB1 cell line (70%), the GB2 cell line (60%), the liver cell line (19%), the intestine cell line (5%) and the lymphocyte cell line (18%) at a concentration $0.665 \times 10^{-8}$ M (Figure 4D). Normal mouse IgM or IgG had an average inhibitory effect of ≤ 10% on gliomas and normal human lines (Figure 4).

### Testing the cytolytic activity of anti-UK MAbs to glioma cells

The ability of anti-UK MAbs to lyse the glioma cells was tested using [$^{3}$H]thymidine release assay, four anti-UK MAbs [MAb UK4 and MAb AB3 (IgM); MAb D4A8 and MAb UK5 (IgG)] and three glioma cell lines (2GB and 1AA). Each lysis experiment was carried out in triplicate. Consistent results were obtained with various glioma cell lines and anti-UK MAbs exhibited efficacies that varied from one MAb to another (Figure 5). AB3 MAb is the most efficient, exerting cytolytic activity at a much lower antibody concentration than the other anti-UK MAbs.

### Testing glioma cells survival after anti-UK MAb treatment

To test whether anti-UK MAbs were able to lyse the glioma cells and not just arrest their proliferation, glioma cells were incubated with anti-UK MAb (AB3 MAb) for 36 h at 37°C as described earlier. Cell survival was examined microscopically after staining with trypan blue. Irrelevant monoclonal antibody of the same isotype and concentration and tissue culture medium was used to monitor the non-specific lysis and spontaneous lysis respectively. Normal human lymphocyte, liver and intestine were treated as glioma and their survival was tested by dye exclusion. Most of the glioma cells retained trypan blue, whereas normal human lymphocyte, liver and intestine cells excluded the dye (data not shown).

### DISCUSSION

Plasminogen activators in tumours of the central nervous system have not been studied extensively despite the intense investigation of their possible role in cancer biology in general (Hoosein et al, 1991; Sumiyoshi et al, 1992; Foekens et al, 1992; Hollas et al, 1992; Kobayashi et al, 1992; Rucklidge, 1992; Hsui et al, 1993; Janicke et al, 1993; Kobayashi et al, 1993; Pujade et al, 1993; Yamashita et al, 1993; Achbarou et al, 1994; Bianchi et al, 1994; Bouchet et al, 1994; Moser et al, 1994; Young et al, 1994). The plasmin generating system is composed of plasminogen, which is a zymogen, and the enzyme plasminogen activator (PA), which activates plasminogen to generate the potent protease plasmin. The high circulating levels of plasminogen represent a reservoir of potential proteolytic activity, which can be recruited by cells for functions requiring localized extracellular proteolysis. The activation of plasminogen is regulated by the amount of PA available; this step is controlled by the cells that secrete the PA. The secretion of PA by cells seems to fluctuate with their physiological or developmental status (Reich, 1978; Strickland, 1980). In the present study glioma tissue showed a very large increase (5 to 44-fold) in UK levels in glioma tissues compared with normal human brain tissues (Figures 1–3). Enhanced production of plasminogen activator has also generally been associated with some cellular responses to tumour promoters (Wigler and Weinstein, 1976), retinoids (Wilson and Reich, 1978) and DNA damage (Miskin and Reich, 1980). High levels of PA have been shown in a wide variety of human tumours (Ossowaski et al, 1973; Wilson and Dowdle, 1979; Wilson and Beck, 1980; Webber et al, 1981; Hoosein et al, 1991; Foekens et al, 1992; Hollas et al, 1992; Kobayashi et al, 1992 Sumiyoski et al 1992; Janicke et al, 1993; Pujade-Lauraine et al, 1993; Yamashita et al, 1993; Achbarou et al, 1994; Bianchi et al, 1994; Bouchet et al, 1994; Moser et al, 1994; Young et al, 1994). The increase in PA levels in these studies varied from 3 to 60-fold, consistent with 5- to 44-fold in our study, when compared with normal tissue.

Plasminogen activators may be involved in growth responses (Kalderon, 1984). They may also represent an essential step in promoting invasiveness and metastasis. These processes require

© Cancer Research Campaign 1998 

that certain cell types transgress the normal anatomical boundaries of tissues and migrate in and out of different body compartments. To allow such cellular migration, mechanisms that provide for the focal degradation must be available. Although the enzymatic basis for such degradation is not completely understood, several lines of evidence have suggested that extracellular proteolysis catalysed by the secretion of plasminogen activators may play an important part in the degradative events necessary for migration of cells in tissues (Reich, 1978). The increased presence of PA may lead to a higher level of both plasmin and collagenase. Plasmin generated by tumour PA not only can destroy the adjacent extacellular matrix, it may also result in normal cell injury and necrosis (Raymond, 1983). Although urokinase-type plasminogen activator plays a central role in cancer invasion and metastasis, uPA is not the only proteolytic enzyme involved in these processes; there are others, such as metalloproteinases, cathepsins, etc. Whether there is a common or different pattern of proteolytic enzymes among various metastatic cancer cells and whether UK-negative cancer cells exist and to what extent is currently under investigation.

An enzyme required for cell migration and tissue destruction, i.e. to control the dissolution of the basement membrane and of the extracellular matrix, should be sitting in the presence of UK on the glioma cell surface. The overexpression of UK and its presence on the outer surface of glioma cells makes UK, therefore, a good marker for immunotherapy of human glioma and tumour metastasis. In this study, the anti-tumorigenic activities of anti-UK MAbs towards human glioma cell lines were evaluated. The results (Figure 4 and Table 1) showed that anti-UK MAbs were able to control the proliferation of glioma cells in vitro. Antibody class seemed to affect the efficacy of the monoclonal antibody. IgM-UK MAbs were more efficient than IgG counterparts. This may be due to the fact that IgM is a pentamer, whereas IgG is a monomer. The cytolytic activities of anti-UK MAbs to glioma cells were then investigated. The results in Figure 5 showed the ability of anti-UK MAbs to lyse glioma cells. The use of these MAbs in vivo for immunotherapy of human gliomas requires the evaluation of their effects on normal cells. The effects of anti-UK MAbs on the three normal cell lines: liver, intestine and lymphocyte were ≤ 20% (Figure 4). At that stage, it is not clear how anti-UK MAbs kill the glioma cells.

Urokinase bound to high-affinity membrane receptor is not readily degraded or endocytosed. Also, the interaction of UK with glioma cells could be considered as that of hormone with its membrane receptor. The region within the A-chain of $M_r$ 55 000 UK, which is homologous to murine epidermal growth factor, has been considered as a growth factor domain that exerts autocrine effects. One possible explanation for the observed antitumorigenic effects of UK MAbs is that binding of anti-UK MAbs to UK-bound receptor may stimulate endocytosis of UK-receptor complex. This may abolish the autocrine effect of the UK-bound receptor and cause glioma cell injury and necrosis. In summary, our results demonstrated that anti-UK MAbs could be a very valuable reagent for cancer immunotherapy and generating anti-metastasis drugs.

### ACKNOWLEDGEMENTS

We thank Dr Joel B Kirkpatrick for the use of his immunofluorescence microscope and photographing equipment, and the Welch Foundation for the award to MZ Atassi of the Robert A Welch Chair of Chemistry.

### ABBREVIATIONS

AA, anaplastic astrocytoma; GB, glioblastoma multiforme; GCS, glioma cell line supernatant; HMW-UK, high molecular weight urokinase; LMW-UK, low molecular weight urokinase; PA, plasminogen activator; uPA, urokinase type plasminogen activator.

### REFERENCES

Abaza MSI and Atassi MZ (1992) Effects of amino acid substitutions outside an antigenic site on protein binding to monoclonal antibodies of predetermined specificity obtained by peptide immunization: demonstration with region 94–100 (antigenic site 3) of myoglobin. *J Prot Chem* **11**: 433

Achbarou A, Kaiser S, Tremblay G, Ste-Marie LG, Brodt P, Goltzman D and Rabbani SA (1994) Urokinase over-production results in increased skeletal metastasis by prostate cancer cells in vivo. *Cancer Res* **54**: 2372–2377

Bianchi E, Cohen RL, Thor AT and Todd RF, Lawrence DA, Ljung BM, Shuman MA and Smith HS (1994) The urokinase receptor is expressed in invasive breast cancer but not in normal breast tissue. *Cancer Res* **54**(4): 861–866

Booth NA, Bennett B, Wijngaards G and Grieve JHK (1983) A new life long hemorrhagic disorder due to excess plasminogen activator. *Blood* **61**: 267

Bouchet C, Spyratos F, Martin PM, Hacene K, Gentile A, and Oglobine J (1994) The prognostic value of urokinase-type plasminogen activator (uPA) and plasminogen activator inhibitors PAI-1 and PAI-2 in breast carcinomas. *Br J Cancer* **69**(2): 398–405

Burnette WN (1981) Electrophoretic transfers of proteins from sodium dodecyl sulfate polyacrylamide gels to unmodified nitrocellulose and radiographic detection with antibody and radioiodinated protein A. *Anal Biochem* **112**: 195

Dano K, Andreasen PA, Grondahl-Hansen J, Kristensen P, Nielsen LS and Skriver L (1986) Plasminogen activators, tissue degradation, and Cancer. *Adv Cancer Res* **44**: 139–266

Duffy MJ (1987) Do proteases play a role in cancer invasion and metastasis? *Eur J Cancer Clin Oncol* **23**: 583–589

Foekens JA, Schmitt M, Van Putten WL, Peters HA, Bontenbal M, Janicke F and Klijn JG (1992) Prognostic value of urokinase-type plasminogen activator in 671 primary breast cancer patients. *Cancer Res* **52**(21): 6101–6105

Gerdin B and Saldeen T (1978) Effect of fibrin degradation products on microvascular permeability. *Thromb Res* **13**: 995

Grimann G, Press H, Schwartze G and Scheurlen PG (1976) Immunosuppression by micromolecular fibrinolysis degradation products in cancer. *Nature* **259**: 399

G-Nzler WA, Steffens GJ, Otting F, Buse G and Flohe L (1982*a*) Structural relationship between human high and low molecular mass urokinase. *Hoppe-Seyler's Z Physiol Chem* **363**: 133

G-Nzler WA, Steffens GJ, Otting F, Kim SM, Frankus E and Floche L (1982*b*) The primary structure of high molecular mass urokinase from human urine. The complete amino acid sequence of the A-chain. *Hoppe-Seyler's Z Physiol Chem* **363**: 1155

Hollas W, Hoosein N, Chung LW, Mazar A, Henkin J, Kariko K, Barnathan ES and Boyd D (1992) Expression of urokinase and its receptor in invasive and non-invasive prostate cancer cell lines. *Thrombosis Haemostasis* **68**(6): 662–666

Hoosein NM, Boyd D, Hollas WJ, Mazar A, Henkin J and Chung LW (1991) Involvement of urokinase and its receptor in the invasiveness of human prostatic carcinoma cell lines. *Cancer Commun* **3**(8): 255–264

Hsui Y, Nishi S, Kitado S and Osada Y (1993) Urokinase-type plasminogen activator antigen as a prognostic factor in bladder cancer. *Nippon Hinyokiko Gakkai Zasshi-Japanese J Urol* **84**(9): 1624–1628

Janicke F, Schmitt M, Pache L, Ulm K, Harbeck N, Hofler H and Graeff H (1993) Urokinase (uPA) and its inhibitor PAI-1 are strong and independent prognostic factors in node-negative breast cancer. *Breast Cancer Res Treat* **24**: 195–208

Kalderon N (1984) Schwann cell proliferation and localized proteolysis: expression of plasminogen-activator predominates in the proliferating cell populations. *Proc Natl Acad Sci USA* **81**: 7216

Kobayashi H, Ohi H, Sugimura M, Shinohara H, Fujii T and Teras T (1992) Inhibition of in vitro ovarian cancer cell invasion by modulation of urokinase-type plasminogen activator and cathepsin B. *Cancer Res* **52**: 3610–3614

Kobayashi H, Moniwa N, Sugimura M, Shinohara H, Ohi H and Terao T (1993) Increased cell-surface urokinase in advanced ovarian cancer. *Jpn J Cancer Res* **84**(6): 633–640

Laemmli UK (1970) Cleavage of structural proteins during the assembly of the head of bacteriophage T4. *Nature* **227**: 680

Miskin R and Reich E (1980) Plasminogen activator: induction of synthesis by DNA damage. *Cell* **19**: 217

*© Cancer Research Campaign 1998*

Moser TL. Young TN. Rodriguez GC. Pizzo SV. Bast RC Jr and Stack MS (1994) Secretion of extracellular matrix-degrading proteinases is increased in epithelial ovarian carcinoma. *Int J Cancer* **56**(4): 552–559
Ng R and Kellen JA (1983) The role of plasminogen activators in metastasis. *Med Hypotheses* **10**: 291
Nielsen LS. Hansen JG. Skriver L. Wilson EL. Kaltoft K. Zeuthen J and Dan K (1982) Purification of zymogen to plasminogen activator from human glioblastoma cells by affinity chromatography with monoclonal antibody. *Biochemistry* **21**: 6410
Ossowski L. Unkeless JC. Tobia A. Quigley JP. Rifkin DB and Reich E (1973) An enzymatic function associated with transformation of fibroblasts by oncogenic viruses (II). *J Exp Med* **137**: 497
Plow EF. Freaney D and Edgnigton TS (1982) Inhibition of lymphocyte protein synthesis by fibrinogen-derived peptides. *J Immunol* **128**: 1595
Pujade-Lauraine E. Lu H. Mirshahi S. Soria J. Soria C. Bernadou A. Kruithof EK. Lijnen HR and Burtin P (1993) The plasminogen-activation system in ovarian tumors. *Int J Cancer* **55**(1): 27–31
Raymond NG and Kellen JA (1983) The role of plasminogen activators in metastasis. *Med Hypoth* **10**: 291
Reich E (1978) Activation of plasminogen: a general mechanism for producing localized extracellular proteolysis. In *Molecular Basis of Biological Degradative Process*. Berlin RD. Herrmann H. Lepow IH and Tanzer JM (eds). pp. 155–169. Academic Press: New York
Reich E (1978) Activation of plasminogen. A widespread mechanism for generating localized extracellular proteolysis. In *Biological Markers of Neoplasia: Basic and Applied Aspects*. Rudden EW (ed). pp. 491–500. Elsevier: New York
Reith A and Rucklidge GJ (1992) Invasion of brain tissue by primary glioma: evidence for the involvement of urokinase-type plasminogen activator as an activator of type IV collagenase. *Biochem Biophys Res Commun* **186**(1): 348–354
Riccio A. Grimaldi G. Verde P. Sebastio G. Boast S and Blasi F (1985) The human urokinase-plasminogen activator gene and its promotor. *Nucleic Acids Res* **13**: 2759
Salerno G. Verde P. Nolli ML. Corti A. Szots H. Meo T. Johnson J. Bullock S. Cassani G and Blasi F (1984) Monoclonal antibodies to human urokinase identify the single-chain pro-urokinase precursor. *Proc Natl Acad Sci USA* **81**: 110
Salo T. Liotta LA. Keshi-Oja J. Turpeenniemi-Hujanen T and Tryggvasson K (1982) Secretion of basement membrane collagen degrading enzyme and plasminogen activator by transformed cells. role in metastasis. *Int J Cancer* **30**: 669
Sobel GW. Mohler SR. Jones NW. Dowdy ABC and Guest MM (1952) Urokinase: an activator of plasma fibrinolysis extracted from urine. *Am J Physiol* **171**: 768
Strickland S (1980) *Development in Mammals*. Vol. 4. Johnson MH (ed.) pp. 81–100. Elsevier: New York
Sumiyoshi K. Serizawa K. Urano T. Takada Y and Baba S (1992) Plasminogen activator system in human breast cancer. *Int J Cancer* **50**(3): 345–348
Towbin H. Staehelin T and Gordon J (1979) Electrophoretic transfer of proteins from polyacrylamide gels to nitrocellulose sheets: procedure and some applications. *Proc Natl Acad Sci USA* **76**: 4350
Verde P. Stoppelli MP. Galeff P. Dinocera PP and Blasi F (1984) Identification and primary sequence of and up spliced human urokinase poly(A⁻) RNA. *Proc Natl Acad Sci* **81**: 4727
Wainberg MA. Israel E and Margolese RG (1982) Further studies on the mitogenic and immune-modulating effects of plasminogen activator. *Immunology* **45**: 715
Webber MM. James G. Lucero L. Van Buskirk JJ and Wettlaufer JN (1981) Plasminogen activator: a marker for human prostatic epithelium and prostate cancer. *In Vitro* **17**: 249
Wigler M and Weinstein IB (1976) Tumor promoter induces plasminogen activator. *Nature* **259**: 232
Wilson EL. Becker MLB. Hoal EG and Dowdle EB (1980) Molecular species of plasminogen activators secreted by normal and neoplastic human cells. *Cancer Res* **40**: 933
Wilson EL and Dowdle E (1979) Secretion of plasminogen activator by normal. reactive and neoplastic human tissues cultured in vitro. *Int J Cancer* **22**: 390
Wilson EL and Reich E (1978) Plasminogen activator in chicken fibroblasts: induction of synthesis by retinoic acid: synergism with viral transformation and phorbol ester. *Cell* **15**: 385
Wun T. Ossowski L and Reich L (1982) A proenzyme form of human urokinase. *J Biol Chem* **257**: 7262
Yamashita J. Ogawa M. Inada K. Yamashita S. Nakashima Y. Saishoji T and Nomura K (1993) Breast cancer prognosis is poor when total plasminogen activator activity is low. *Br J Cancer* **67**(2): 374–378
Young TN. Rodriguez GC. Moser T. Bast Jr. R. Pizzo SV and Stack MS (1994) Coordinate expression of urinary type plasminogen activator and its receptor accompanies malignant transformation of the ovarian surface epithelium. *Am J Obstetrics Gynecol* **170**(5pt1): 1285–1296
Zucker SA (1988) Critical appraisal of the role of proteolytic enzymes in cancer invasion: emphasis on tumor surface proteinases. *Cancer Invest* **6**: 219–231

© Cancer Research Campaign 1998